# *Rubroshiraia* gen. nov., a second hypocrellin-producing genus in Shiraiaceae (Pleosporales)

**DOI:** 10.3897/mycokeys.58.36723

**Published:** 2019-08-28

**Authors:** Dong-Qin Dai, Nalin N. Wijayawardene, Li-Zhou Tang, Chao Liu, Li-Hong Han, Hong-Long Chu, Hai-Bo Wang, Chun-Fang Liao, Er-Fu Yang, Rui-Fang Xu, Yun-Min Li, Kevin D. Hyde, D. Jayarama Bhat, Paul F. Cannon

**Affiliations:** 1 Center for Yunnan Plateau Biological Resources Protection and Utilization, College of Biological Resource and Food Engineering, Qujing Normal University, Qujing, Yunnan 655011, China Qujing Normal University Qujing China; 2 State Key Laboratory of Genetic Resources and Evolution, Kunming Institute of Zoology, Chinese Academy of Sciences, Kunming, Yunnan 650223, China Kunming Institute of Zoology, Chinese Academy of Sciences Kunming China; 3 Centre of Excellence in Fungal Research, Mae Fah Luang University, Chiang Rai 57100, Thailand Mae Fah Luang University Chiang Rai Thailand; 4 No. 128/1-J, Azad Housing Society, Curca, P.O. Goa Velha 403108, India Unaffiliated Goa India; 5 Royal Botanic Gardens, Kew, Surrey TW9 3AB, UK Royal Botanic Gardens Kew United Kingdom

**Keywords:** HPLC, metabolite, new genus, phylogeny, taxonomy

## Abstract

Shiraiaceae is an important family in Pleosporales (Dothideomycetes), which includes medical fungi and plant pathogens. Two hypocrellin-producing taxa, *Shiraia bambusicola* and a novel genus *Rubroshiraia***gen. nov.**, typified by *Rubroshiraia bambusae* are treated in this article. Maximum likelihood analysis, generated via RAxML (GTR+G model), using a combined SSU, LSU, TEF1 and RPB2 sequence dataset, shows that *Rubroshiraia* is close to *Shiraia* and belongs to the family Shiraiaceae. Descriptions, illustrations and a taxonomic key are provided for the genera in Shiraiaceae. *Rubroshiraia* morphologically differs from *Shiraia* in having small and dark ascostromata and filiform ascospores. Production of the ascostromatal metabolites, hypocrellin A and B, were examined by HPLC and spectrophotometer. The content of hypocrellin A and B of specimen HKAS 102255 (*R. bambusae*) is twice that produced by HKAS 102253 (*S. bambusicola*). To clarify the relationship between *R. bambusae* and *Hypocrella bambusae*, type material of the latter was examined and provided the illustration.

## Introduction

Liu et al. (2013) introduced the family Shiraiaceae Y.X. Liu, Zi Y. Liu & K.D. Hyde which is typified by *Shiraia* Henn. and placed the family in Pleosporales Luttr. ex M.E. Barr. Ariyawansa et al. (2013) accommodated *Grandigallia* M.E. Barr, Hanlin, Cedeño, Parra & R. Hern. in Shiraiaceae since it morphologically resembles *Shiraia*. Subsequent publications by Wijayawardene et al. (2014, 2017, 2018) agreed with this placement and, thus, the family currently comprises two genera.

*Shiraia* is typified by *S. bambusicola* Henn. (Hennings 1900), which is parasitic on living bamboo culms and has conspicuous large, pinkish, fleshy ascostromata with multi-locules located near the periphery, fissitunicate asci and hyaline, muriform ascospores (Liu et al. 2013). *S. bambusicola* has been reported from temperate regions of Asia, such as China and Japan (Table 1) (Hino 1961; Li et al. 2009; Liu et al. 2013).

**Table 1.** Distribution of *Shiraia bambusicola*.

Distribution		References
Country	Province	
China	Anhui	Li et al. (2009), Lai and Fu (2000)
	Guangxi	Li et al. (2009)
	Guizhou	Li et al. (2009)
	Henan	Li et al. (2009)
	Hubei	Li et al. (2009)
	Hunan	Li et al. (2009)
	Jiangsu	Zhao and Liang (2005), Li et al. (2009)
	Jiangxi	Li et al. (2009)
	Sichuan	Chen and Chen (2009), Li et al. (2009)
	Yunan	Fang et al. (2006), Chen et al. (2010)
	Zhejiang	Li et al. (2009), Liu et al. (2013)
Japan	Tokyo	Hino (1961), Liu et al. (2013)
	Osaka	Morakotkarn et al. (2007)

*Shiraia* has previously been placed in several families, depending on the opinions of authors. Hennings (1900) considered *Shiraia* to have unitunicate asci and treated as a member in the family Nectriaceae Tul. & C. Tul. (Hypocreales, Sordariomycetes) when he established the genus. Based on its large and fleshy fruiting bodies, *Shiraia* was transferred to Hypocreaceae De Not by Saccardo (1902). Amano (1980) re-examined the type specimen and regarded *Shiraia* as having bitunicate asci and, hence, placed the genus in Pleosporaceae Nitschke (Pleosporales, Dothideomycetes). However, it was subsequently transferred to Dothideales, genera *incertae sedis* by Kirk et al. (2001).

Earlier classifications of *Shiraia* were based on morphological characters. The first attempt of DNA-based taxonomy (Cheng et al. 2004) confirmed that *Shiraia* belongs in Pleosporales and was phylogenetically close to species of Phaeosphaeriaceae M.E. Barr. Thus, Cheng et al. (2004) considered *Shiraia* as a member in Phaeosphaeriaceae. Liu et al. (2013) carried out significant studies on *Shiraia* taxonomy by re-examining the holotype and carrying out phylogenetic analysis, based on LSU sequence data. Liu et al. (2013) also designated an epitype of both sexual and asexual morphs and introduced Shiraiaceae in the Pleosporales.

*Shiraia bambusicola* has been reported as a pathogen on various bamboo species (Table 2) or as endophyte of bamboo culms (Morakotkarn et al. 2007, 2008). The bamboo genus *Brachystachyum* Keng is significantly affected by *S. bambusicola* (Table 2; Lai and Fu 2000). The holotype of *S. bambusicola* was recorded from a *Bambusa* sp. (Liu et al. 2013). *Shiraia bambusicola* has also been recorded on several common bamboo genera, including *Fargesia* Franch., *Phyllostachys* Sieb. et Zucc., *Pleioblastus* Nakai and *Indosasa* Mcclure (Lai and Fu 2000; Li et al. 2009). However, these hosts need to be further verified.

**Table 2.** List of bamboo hosts of *Shiraia bambusicola*.

Bamboo host	References
*Brachystachyum densiflorum* (Rendle) Keng	Lai and Fu (2000)
*Brachystachyum albostriatum* G.H. Lai	Li et al. (2009)
*Brachystachyum ensiflorum* (Pendle) Keng	Li et al. (2009)
*Brachystachyum yixingense*	Li et al. (2009)
*Phyllostachys nidularia* Munro	GenBank
Phyllostachys praecox f. prevernalis S.Y. Chen & C.Y. Yao	GenBank
*Pleioblastus amarus* (Keng) Keng f.	GenBank

*Shiraia bambusicola* produces hypocrellins. Four hypocrellins have been extracted from the fungal stromata (Wan and Chen 1981; Kishi et al. 1991; Chen and Chen 2009). Endophytes, named as *Shiraia* spp., were also shown to produce hypocrellins on media (Lu et al. 2004; Morakotkarn et al. 2008; Liang et al. 2009; Zhang et al. 2014; Tong et al. 2017). The fruiting body of “Zhuhongjun” also contains hypocrellins (Hudson et al. 1994; Huang et al. 2001). Hypocrellin seems to be an important feature when clarifying the taxa of Shiraiaceae.

A Chinese medical fungus named “Zhuhongjun” in Chinese, was identified as *Hypocrella bambusae* (Berk. & Broome) Sacc. by Liu (1978), based on its conspicuous and fleshy fruiting body. However, according to our knowledge, Zhuhongjun is similar to *S. bambusicola* and unrelated to *Hypocrella*. Therefore, the taxonomic status of this taxon needs to be clarified.

The monotypic genus *Grandigallia*, collected on *Polylepis sericea* Wedd. (*Rosaceae*), was introduced by Barr et al. (1987) with *G. dictyospora* M.E. Barr et al. as the type species. *Grandigallia dictyospora* was reported from Venezuela in a locality above 3,400 m and the fungus was found to produce large ascostromata (3–14 cm in diam.), with bitunicate asci and dictyospores (Barr et al. 1987).

In this study, ten specimens of *S. bambusicola* and a hypocrellin producing taxon (“Zhuhongjun” in Chinese) were collected from Yunnan Province in China. Morphological and phylogenetic studies were carried out to determine the taxonomic status of these taxa. Sequences from endophytic strains, named as *Shiraia* spp., were also downloaded from GenBank and included in the phylogenetic analyses. The metabolite content of hypocrellin extracted from the specimens was determined by HPLC (Chem 2012). Based on the morphology and phylogenetic analyses, the hypocrellin producing taxon “Zhuhongjun” is treated as a new genus in Shiraiaceae.

## Material and methods

### Collecting and examination of specimens

Bamboo culms with large, reddish to pale yellow ascostromata were collected from Yunnan, China and brought to the laboratory in 2017. Samples were examined following the methods described in Dai et al. (2017). Micro-morphological characters were examined and photographed by differential interference contrast (DIC), using a Leica DM2500 compound microscope with a Leica DMC4500 camera. Fruiting bodies were observed by stereomicroscopy using a Leica S8AP0 and photographed by HDMI 200C. Measurements were made using Tarosoft (R) Image Frame Work software. Specimens have been deposited at the herbarium of Kunming Institute of Botany, Chinese Academy of Sciences (**KUN**) and Herbarium Mycologicum, Academiae Sinicae (**HMAS**) in Beijing. Facesoffungi (Jayasiri et al. 2015) and Index Fungorum (Index Fungorum 2019) numbers were provided for new taxa. Type material of *H. bambusae* was loaned and examined from the Royal Botanic Gardens, Kew.

### DNA extraction, PCR amplification and sequencing

The surface of fungal fruiting bodies was sterilised by 75% alcohol and rinsed three times in sterile water. The internal tissue with locules was cut into pieces and ground in a mortar into powder with liquid nitrogen. The powder was used to directly extract DNA with an OMEGA E.Z.N.A. Forensic DNA Kit, following the manufacturer’s instructions.

ITS5 and ITS4, NS1 and NS4 (White et al. 1990) and LROR and LR5 (Vilgalys and Hester 1990) primers were used for the amplification of internal transcribed spacers (ITS), small subunit rDNA (SSU) and large subunit rDNA (LSU), respectively. Translation elongation factor 1-α gene region (TEF 1-alpha) and RNA polymerase II second largest subunit (RPB2) genes were amplified by using EF1-983F and EF1-2218R (Rehner 2001), fRPB2-5f and fRPB2-7cr primers (Liu et al. 1999), respectively.

The final volume of the polymerase chain reaction (PCR) was prepared following Dai et al. (2017). The PCR thermal cycle programme of ITS, SSU, LSU, RPB2 and TEF 1-alpha genes amplifications were run under the same conditions as described in Dai et al. (2017). The quality of PCR products was checked by 1% Biowest agarose gel electrophoresis. Amplified PCR fragments were sequenced at Shanghai Majorbio Bio-Pharm Technology Co., Ltd. and BGI Tech Solutions Co., Ltd. (BGI-Tech), P.R. China. Generated new sequences of ITS, LSU, SSU, Rpb2 and TEF1 regions are deposited in GenBank (Table 4).

### Phylogenetic analysis

The BLAST searches in GenBank, using LSU and ITS sequence data were carried out to obtain the close strains. Additional sequences were downloaded from GenBank based on recent publications (Liu et al. 2017).

Single gene sequence alignments were carried out with MAFFT v. 7.215 (Katoh and Standley 2013, http://mafft.cbrc.jp/alignment/server/index.html) and edited manually when necessary in BioEdit v. 7.0 (Hall 2004). The alignments of LSU, SSU, Rpb2 and TEF1 regions were combined in MEGA6 version 6.0 (Tamura et al. 2013).

Maximum-likelihood (ML) analyses, including 1000 bootstrap replicates, were run using RAxMLGUI v.1.0. (Stamatakis 2006; Silvestro and Michalak 2011). Alignments in PHYLIP format were exchanged and loaded from the website (http://sing.ei.uvigo.es/ALTER/). The online tool Findmodel (http://www.hiv.lanl.gov/content/sequence/findmodel/findmodel.html) was used to determine the best nucleotide substitution model for each partition data.

Maximum-parsimony (MP) analyses were carried out in PAUP v. 4.0b10 (Swofford 2002) with 1000 replications. Maxtrees were set to 1000, branches of zero length were collapsed and all multiple equally most parsimonious trees were saved. The robustness of the most parsimonious trees was evaluated from 1000 bootstrap replications (Phillips et al. 2013).

Bayesian analyses were performed using MrBayes v. 3.0b4 (Ronquist and Huelsenbeck 2003). The model of evolution was performed using MrModeltest v. 2.2 (Nylander 2004). Posterior Probabilities (PP) (Rannala and Yang 1996; Zhaxybayeva and Gogarten 2002) were determined by Markov Chain Monte Carlo sampling (MCMC) in MrBayes v. 3.0b4 (Huelsenbeck and Ronquist 2001). Six simultaneous Markov chains were run for 1,000,000 generations and trees were sampled every 100^th^ generation. The burn-in was set to 0.25 and the run was automatically stopped when the average standard deviation of split frequencies reached below 0.01 (Maharachchikumbura et al. 2015).

Trees were visualised with TreeView (Page 1996) or FigTree v. 1.4.0 (http://tree.bio.ed.ac.uk/software/figtree/) and, additionally, layouts were done with Adobe Illustrator CS v. 5. Maximum-likelihood bootstrap values (MLBP) and Maximum-parsimony bootstrap values (MPBP) equal to or greater than 50% are given for each tree. Bayesian posterior probabilities (BYPP) > 0.90 are indicated as thickened lines. The sequences used in this study are listed in Table 1. The combined alignment and phylogenetic tree were submitted at TreeBASE (http://purl.org/phylo/treebase/phylows/study/TB2:S24345).

### HPLC profiling

**Figure 1.**
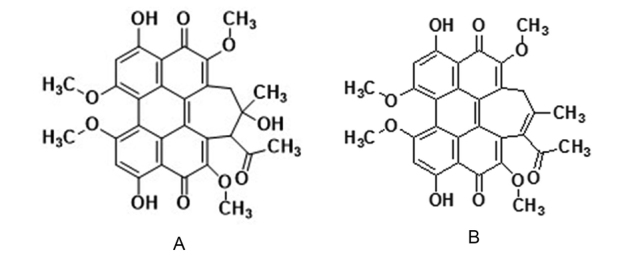
Chemical structures of hypocrellin A and hypocrellin B. **A** hypocrellin A **B** hypocrellin B.

Standards of hypocrellin A and hypocrellin B were purchased from Shanghai Tauto Biotech CO., Ltd. (http://www.tautobiotech.com) and used as received. Their purity is ≥ 98% (HPLC) and their structures are redrawn based on references (Wan and Chen 1981; Morakotkarn et al. 2008) and shown in Figure 1. The dry powder of ascostromata of *S. bambusicola* (HKAS102266) and “Zhuhongjun” (HKAS102270) was extracted followed the methods described by Stadler et al. (2001) and accurately weighed to 0.5 g and added to 25 ml of methanol and sonicated for 30 min. Semi-preparative HPLC was performed on an Agilent 1260 apparatus equipped with a UV detector and a CAPCELL PAK C18 (Agilent, 4.6 mm × 25 cm, 5 µm) column, with 38% solvent A: H_2_O + 0.5% formic acid; 62% solvent B: acetonitrile, isocratic elution, UV/Vis the detection in the range of 265 nm (Table 3). The UV-Vis spectra were recorded at room temperature on a Perkin-Elmer Lambda 900 spectrophotometer (Fig. 5).

**Table 3.** HPLC condition used in this study.

Instrument	Condition
Reverse phase-column	CAPCELL PAK C18 (4.6 mm × 25 cm, 5 µm)
Oven temp. (°C)	35
Flow rate (ml/min)	1
Mobile phase (%)	38% solvent A: H2O + 0.5% formic acid; 62% solvent B: acetonitrile
UV Absorbance (nm)	265
Gradient elution	isocratic elution
Run time (min)	30–40

**Table 4.** List of newly generated sequences with their culture collection numbers and GenBank accession numbers.

Organism	Specimen voucher	GenBank accession numbers				
		ITS	LSU	SSU	TEF	RPB2
*Shiraia bambusicola*	HKAS102253	MK804668	MK804648	MK804694	MK819208	MK819228
	HKAS102254	MK804669	MK804649	MK804695	MK819209	MK819229
	HKAS102257	MK804670	MK804650	MK804696	MK819210	MK819230
	HKAS102261	MK804671	MK804651	MK804697	MK819211	MK819231
	HKAS102262	MK804672	MK804652	MK804698	MK819212	MK819232
	HKAS102263	MK804673	MK804653	MK804699	MK819213	MK819233
	HKAS102264	MK804674	MK804654	MK804700	MK819214	MK819234
	HKAS102265	MK804675	MK804655	MK804701	MK819215	MK819235
	HKAS102266	MK804676	MK804656	MK804702	MK819216	MK819236
	HKAS102267	MK804677	MK804657	MK804703	MK819217	MK819237
*Rubroshiraia bambusae*	**HKAS102255**	**MK804678**	**MK804658**	**MK804704**	**MK819218**	
	HKAS102256	MK804679	MK804659	MK804705	MK819219	
	HKAS102260	MK804680	MK804660	MK804706	MK819220	
	HKAS102268	MK804681	MK804661	MK804707	MK819221	
	HKAS102269	MK804682	MK804662	MK804708	MK819222	
	HKAS102270	MK804683	MK804663	MK804709	MK819223	
	HKAS102271	MK804684	MK804664	MK804710	MK819224	
	HKAS102272	MK804685	MK804665	MK804711	MK819225	
	HKAS102273	MK804686	MK804666	MK804712	MK819226	
	HKAS102274	MK804687	MK804667	MK804713	MK819227	

The holotype specimen is highlighted in bold. Abbreviations: HKAS: herbarium of Kunming Institute of Botany, Chinese Academy of Sciences.

## Results

### Phylogeny

**Figure 2.**
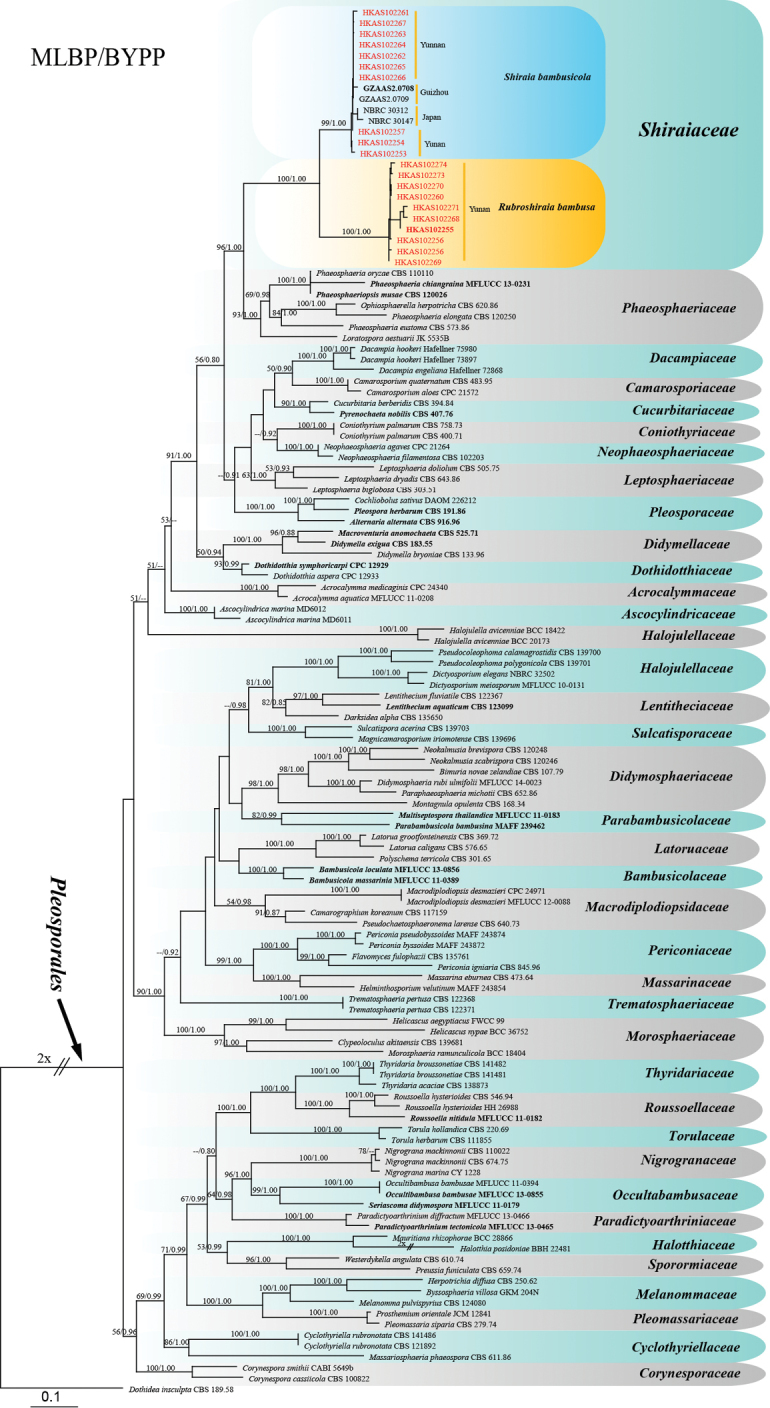
Maximum likelihood phylogenetic tree generated from RAxML (GTR+G model), based on combined LSU, SSU, TEF1 and RPB2 sequences data. ML values (MLBP) (> 50%), resulting from 1000 bootstrap replicates and Bayesian posterior probabilities (BYPP) greater than 0.90, are given at the nodes. The original isolate numbers’ codes are noted after the species names. The tree is rooted to *Dothidea insculpta* (CBS 189.58). Ex-type or ex-epitype strains are in bold. Newly generated strains are in *red* and the new genus is in yellow background.

To clarify the family placement of newly established taxa, maximum likelihood phylogenetic analysis was generated from RAxML (GTR+G model), based on combined SSU, LSU, TEF1 and RPB2 sequences data (Fig. 2). The combined alignment comprised 4025 characters including gaps for 127 ingroup taxa and one outgroup taxon *Dothidea insculpta* (CBS 189.58). Based on the phylogenetic tree in Fig. 2, the new collections cluster within family Shiraiaceae with high bootstrap support (96/1.00 MLBS/BSPP) and emerge as two groups, which are *S. bambusicola* lineage and a new clade named as *R. bambusae* in this paper. *Shiraia* and *Rubroshiraia* have more or less similar ascostromata and both of them can produce the metabolite hypocrellins. However, they can be phylogenetically distinguished with high bootstrap support (100/1.00 MLBS/BSPP) (Fig. 2). *Grandigallia* has not been included in phylogenetic analysis as it is lacking sequences in the GenBank. However, the new taxa can be morphologically distinguished from it. Shiraiaceae is phylogenetically close with family Phaeosphaeriaceae in Pleosporales and this has been confirmed by Liu et al. (2013).

**Figure 3.**
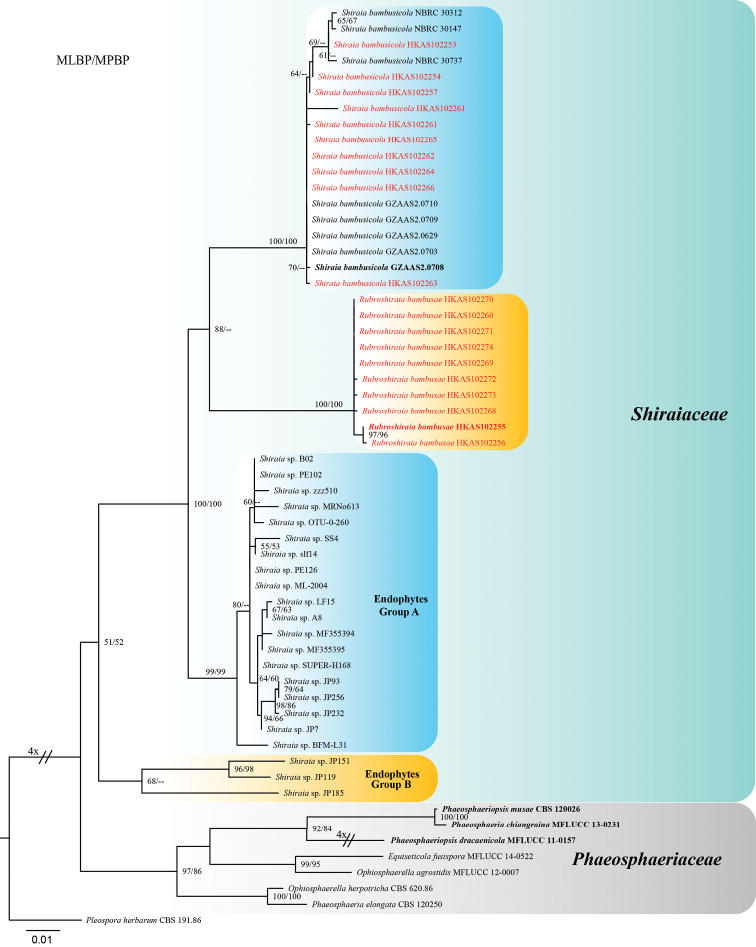
Maximum likelihood phylogenetic tree generated from RAxML (GTR+G model), based on combined LSU and ITS sequences data. ML and MP values (MLBP/MPBP) (> 50%), resulting from 1000 bootstrap replicates, are given at the nodes. The original isolate numbers’ codes are noted after the species names. The tree is rooted to *Pleospora herbarum* (CBS 191.86). Ex-type or ex-epitype strains are in bold. Newly generated strains are in red.

To clarify the relationship between endophytic strains named as shiraia-like (*Shiraia* spp.) and Shiraiaceae, a phylogenetic tree was constructed (RAxML (GTR+G model), based on combined LSU and ITS sequences data and compared. The combined alignment comprises 1442 characters including gaps for 57 ingroup taxa and one outgroup taxon *Pleospora herbarum* (CBS 191.86). Of the 1442 characters of the combined matrix, 1116 were constant and 220 were parsimony informative. The endophytic strains separated into two lineages (Group A and group B) forming at the base clade of Shiraiaceae (Fig. 3). Several strains in group A ca. JP7, JP93, JP232, JP256, SUPER-H168, A8 and ML-2004, isolated from bamboo tissue can produce hypocrellins in media (Lu et al. 2004; Morakotkarn et al. 2008; Liang et al. 2009; Cai et al. 2011; Zhang et al. 2014). However, no hypocrellins were detected from Group B, which included three Japanese strains viz. JP119, JP151 and JP185 (Morakotkarn et al. 2008).

### Metabolites production

**Figure 4.**
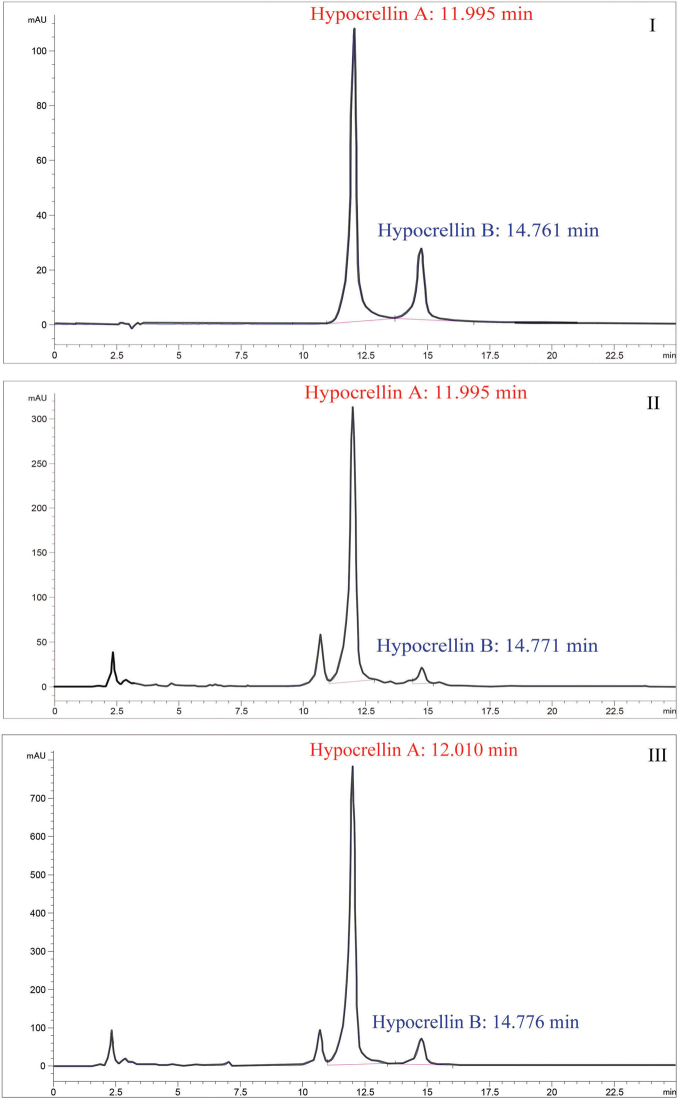
Hypocrellin A and hypocrellin B HPLC-UV profiles (265 nm) of standards and stromatal HPLC-UV profiles (265 nm) of specimens of *Shiraia bambusicola* (HKAS 102253) (II) and *Rubroshiraia bambusae* (HKAS 102255) (III) and DAD spectra of major metabolites.

**Figure 5.**
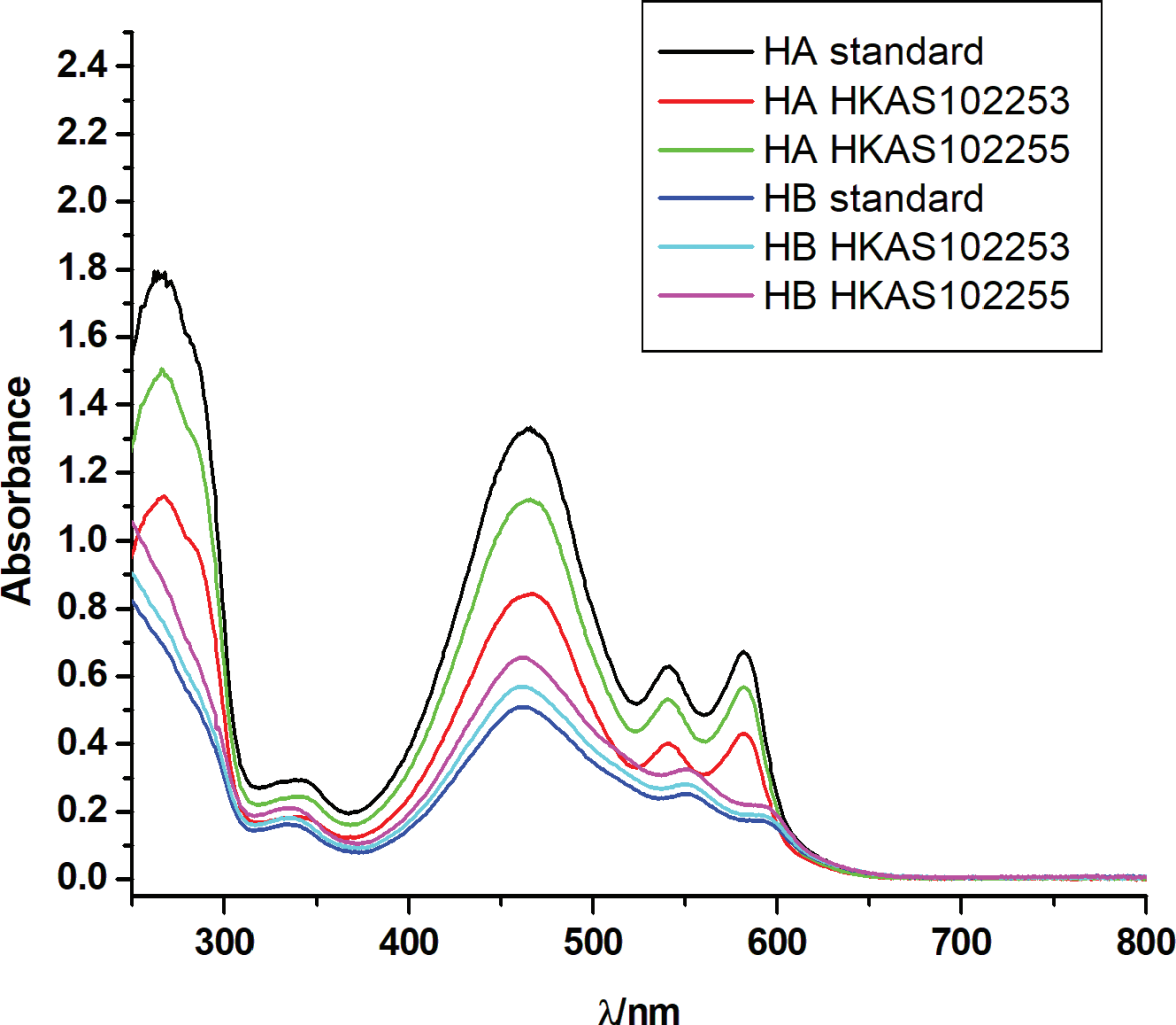
The UV spectrum of the standards and of hypocrellin A and B from the samples (*Shiraia bambusicola*HKAS 102253 and *Rubroshiraia bambusae*HKAS 102255) were recorded in alcohol at room temperature. HA: hypocrellin A, HB: hypocrellin B.

Stromatal extracts from specimens of *S. bambusicola* (HKAS102266) and *R. bambusae* (HKAS102270) contained high quantities of hypocrellin A (304.03 ng/ul and 790.86 ng/ul, respectively). Stromatal extracts from specimens of *S. bambusicola* contained 42.55 ng/ul hypocrellin B, whereas *R. bambusae* produces a higher quantity (204.60 ng/ul). The HPLC profiles of *S. bambusicola* and *R. bambusae* are depicted in Figure 4. The UV spectrum of the standards and of hypocrellin A and B from the samples (*S. bambusicola*HKAS 102253 and *R. bambusae*HKAS 102255) were recorded in alcohol and shown in Figure 5.

## Taxonomy

### Shiraiaceae

Taxon classificationFungiPleosporalesShiraiaceae

Y.X. Liu, Zi Y. Liu & K.D. Hyde, Phytotaxa 103(1): 53 (2013)

FE829B88F4F85051A601B10DCC0C5256

#### Notes.

The family Shiraiaceae was introduced by Liu et al. (2013) with a single genus and later Grandigallia was added to this family by Ariyawansa et al. (2013). In previous studies, Shiraiaceae was closely related with Phaeosphaeriaceae and their distinction was questionable (Cheng et al. 2004, Liu et al. 2013). However, our multi-gene analyses (Fig. 2) clearly indicate that Shiraiaceae and Phaeosphaeriaceae are distinct. Evidence is also borne out by the fact the Phaeosphaeriaceae have single ascostromata (Phookamsak et al. 2014), while in Shiraiaceae, ascostromata have multiple ascomata. Moreover, Shiraiaceae produces a high quantity of hypocrellins and no such metabolites, secreted by Phaeosphaeriaceae, were reported as far as we know (Phookamsak et al. 2014). In this study, the third genus (i.e. Rubroshiraia) is introduced to the family and produces hypocrellins. The endophytic strains in the phylogenetic tree in Figure (2) probably can be named as new genera, once the types are selected. Thus, currently three genera are placed in Shiraiaceae.

#### Type genus.

Shiraia Henn., Bot. Jb. 28(3): 274 (1900).

#### Type species.

S. bambusicola Henn., Bot. Jb. 28(3): 274 (1900).

### Shiraia bambusicola

Taxon classificationFungiPleosporalesShiraiaceae

Henn., Bot. Jb. 28(3): 274 (1900)

981E92E478AF50D18ABCF92E6A692755

Fig. 6

#### Description.

**Parasitic** on living branches of bamboo. **Sexual morph**: ***Ascostromata*** 1–6 cm long × 1–4 cm wide, solitary, superficial, subglobose, long ellipsoid to irregular, tuberculate, fleshy, white to pinkish, with locules lining the periphery, with dark ostiolate points appearing on surface. ***Ascostromatic tissue*** thick, pinkish, composed of wide, woven hyphae of textura intricata. ***Locules*** in vertical section 370–700 µm high × 370–700 µm diam. (*x̄* = 541 × 513 µm, n = 20), globose to subglobose, immersed in the peripheral layer of ascostromata, with 100–200 µm wide ostioles. ***Peridium*** 20–45 µm thick, composed of several layers of hyaline to light brown, small cells of textura angularis to textura intricata. ***Hamathecium*** composed of interthecial, hyaline septate, branched pseudoparaphyses, 1–2.5 µm wide. ***Asci*** 200–370 × 20–35 µm (*x̄* = 291.6 × 26.6 µm, n = 20), 4–6-spored, thick-walled, bitunicate, fissitunicate, cylindrical, short-pedicellate, with an ocular chamber. ***Ascospores*** 50–77 × 15–24 µm (*x̄* = 62.3 × 18.1 µm, n = 20), 1-seriate, overlapped, fusiform, muriform, hyaline, with 7 transverse septa, constricted at the septum, smooth-walled. **Asexual morph**: ***Conidiomata*** 200–500 µm high, 300–400 µm wide, loculate, forming within ascostromata, globose to subglobose or irregular. ***Wall of locules*** 20–40 µm thick, composed of several layers of hyaline to light brown, small cells of textura intricata. ***Conidiophores*** reduced to conidiogenous cells. ***Conidiogenous cells*** 3–6 × 2–3 µm (*x̄* = 4.7 × 2.1 µm, n = 10), blastic, cylindrical, hyaline, smooth-walled. ***Conidia*** 60–80 × 19–25 µm (*x̄* = 75.4 × 23.1 µm, n = 20), fusiform, muriform, hyaline, with irregularly transverse and longitudinal septa, straight to curved, smooth-walled.

**Figure 6.**
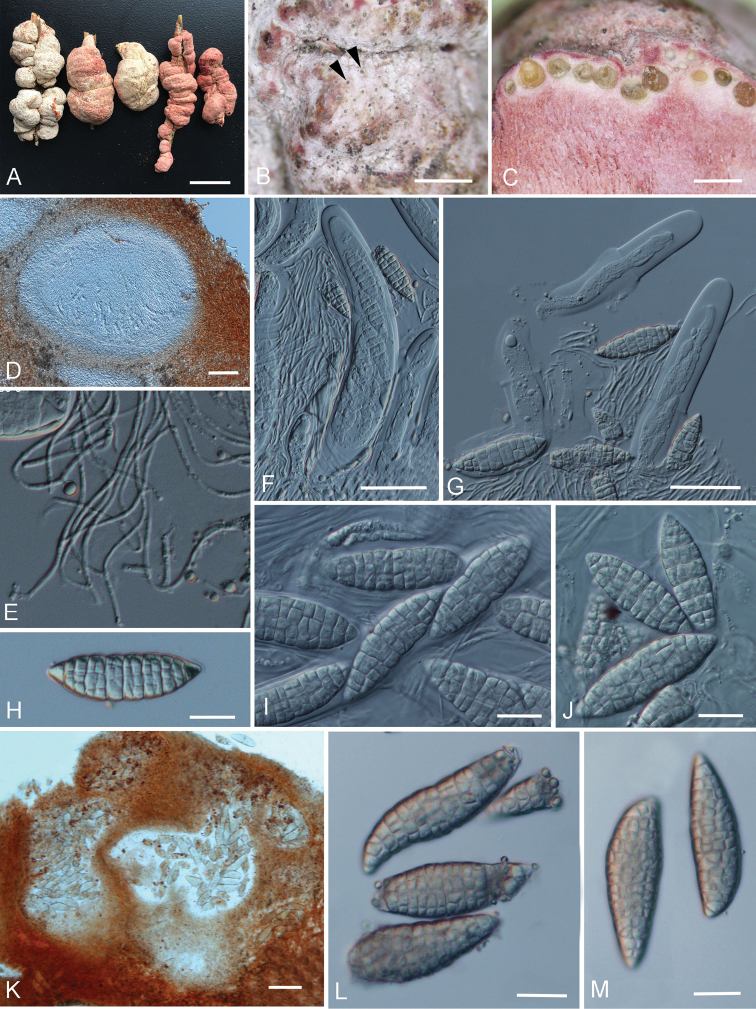
*Shiraia bambusicola***A–J** sexual morph **A** fruiting bodies (HKAS102253, HKAS102254, HKAS102257, HKAS102261, HKAS102262) **B–J** photographs from material HKAS102253 **B** Surface of ascostromata showing the dark openings of ostiole **C** vertical section of ascostromata **D** vertical section of locule **E** pseudoparaphyses **F, G** asci (**G** Showing the fissitunicate asci) **H–J** ascospores **K–M** asexual morph **K** vertical section of asexual locules **L–M** conidia. Scale bars: 2 cm (**A**), 5 mm (**B**), 1 mm (**C**), 100 μm (**D, K**), 50 μm (**F, G**), 20 μm (**H–J, L, M**).

#### Culture characters.

Colonies growing slowly, attaining 30 mm diam. after 2 weeks at 27 °C under dark, circular, with even margin, floccose at the centre, drift white at margin, light greenish at centre, dark from below.

#### Material examined.

CHINA, Yunnan province, Lijiang, on living branches of *Brachystachyum densiflorum* (Rendle) Keng, 3 May 2017, Dong-Qin Dai, DDQ00409 (HKAS102253), *Ibid*. (duplicate specimen deposited in HMAS 290446), *Ibid*. DDQ00410 (HKAS102254), *Ibid*. DDQ00413 (HKAS102257), *Ibid*. 10 June 2017, Dong-Qin Dai, DDQ00418 (HKAS102261), *Ibid*. DDQ00419 (HKAS102262), *Ibid*. DDQ00420 (HKAS102263), *Ibid*. DDQ00421 (HKAS102264), *Ibid*. DDQ00422 (HKAS102265), *Ibid*. DDQ00423 (HKAS102266), *Ibid*. DDQ00424 (HKAS102267).

#### Notes.

*Shiraia bambusicola* was erected by Hennings (1900), based on a collection from Japan. Liu et al. (2013) re-examined the holotype with 1–2.5 cm wide ascostromata, which is smaller than the new collections (1–4 cm wide in ascostromata) in China. The holotype has large ascospores compared with the new specimens in this study (75–125 × 23–47 µm vs. 50–77 × 15–24 µm). The epitype designated by Liu et al. (2013) which has similar-sized (50–77 × 15–24 µm) ascospores and similar ITS sequence, as in our new collections.

##### Other genera included

### Grandigallia

Taxon classificationFungiPleosporalesShiraiaceae

M.E. Barr et al., Mycotaxon 29: 196. 1987.

779F11C3FA33521CA77BAA085CE562DD

#### Description.

See Ariyawansa et al. (2013).

#### Type species.

*Grandigallia dictyospora* M.E. Barr et al., Mycotaxon 29: 196 (1987)

#### Notes.

The monotypic genus *Grandigallia* was introduced by Barr (1987) and is typified by *G. dictyospora*. The fungus infects branches of *Polylepis sericea* Wedd. (*Rosaceae*) and produces conspicuous (3–14 cm in diam.) and black ascostromata. *Grandigallia* closely resembles *Shiraia* in having muriform ascospores, however, it differs by its black and larger ascostromata. Kirk et al. (2008) and Lumbsch and Huhndorf (2010) placed *Grandigallia* in Dothideomycetes, genera *incertae sedis.*Ariyawansa et al. (2013) re-examined the type material and transferred it to Shiraiaceae in Pleosporales. Wijayawardene et al. (2014, 2017, 2018) accepted this placement.

### Rubroshiraia

Taxon classificationFungiPleosporalesShiraiaceae

D.Q. Dai & K.D. Hyde gen. nov.

D006E91FAED454F682DB6F5C97D99A97

#### Etymology.

The epithet “*Rubro*” means red colour referring to reddish ascotromata similar to the genus *Shiraia*.

#### Description.

**Parasitic** on living branches of bamboo. **Sexual morph**: ***Ascostromata*** solitary, superficial, globose to subglobose, fleshy, reddish, with locules lining the periphery, with dark ostiolate tips appearing on surface. ***Ascostromatic tissue*** thick, pinkish, composed of wider woven hyphae of textura intricata. ***Locules*** globose to subglobose, immersed in the peripheral layer of ascostromata, with narrow ostiolate openings. ***Peridium*** composed of several layers of hyaline to dark brown, small cells of textura angularis to textura intricata. ***Hamathecium*** of interthecial, hyaline, septate, branched pseudoparaphyses above asci. ***Asci*** 8-spored, thick-walled, bitunicate, fissitunicate, cylindrical, short-pedicellate, with an ocular chamber. ***Ascospores*** spirally arranged in asci, filiform, hyaline, with transverse septa, smooth-walled. **Asexual morph**: Undetermined.

#### Type species.

*R. bambusae* D.Q. Dai & K.D. Hyde.

#### Notes.

The hypocrellin-producing fungus *R. bambusae* is a well-known taxon used in Chinese traditional medicine which is called “Zhuhongjun” or “Zhuxiaorouzhuojun” in Chinese. However, without molecular data, it was wrongly named as *H. bambusae* (Liu 1978).

**Figure 7.**
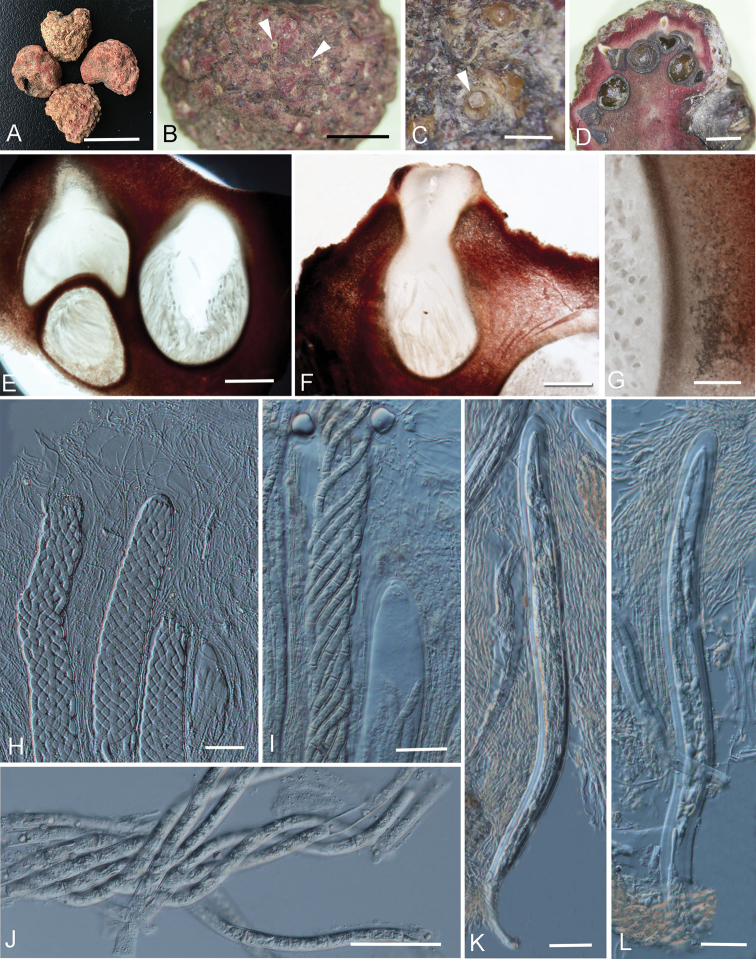
*Rubroshiraia bambusae* (HKAS102255, holotype) **A** fruiting bodies **B, C** surface of ascostromata showing the openings of ostiole **D** vertical section of ascostromata **E, F** vertical section of locule **G** peridium of locule **H** asci and pseudoparaphyses **I** asci and asci ocular chamber **J** ascospores **K, L** immature asci. Scale bars: 1 cm (**A**), 25 mm (**B**), 2 mm (**C, D**), 500 μm (**E, F**), 200 μm (**G**), 50 μm (**H–L**).

*Hypocrella bambusae* was combined by Saccardo (1878), based on its linear asci and filiform ascospores. Index Fungorum (2019) lists its basionym as *Hypocrea bambusae* Berk. & Broome, which was collected on the inflorescences of bamboo in Sir Lanka and had linear asci and filiform ascospores (Berkeley and Broome 1875). Liu (1978) recorded a well-known Chinese medicinal ascomycete, producing 0.7–1.5 mm diam., hemispheric and reddish stromata with multi-locules, cylindrical asci and filiform ascospores which are spirally arranged and more than 250 μm long on bamboo culms. Liu (1978) identified this fungus as *H. bambusae*, probably based on its cylindrical asci and filiform ascospores. In addition, species of *Hypocrella* usually produce perithecial ascomata (Saccardo 1878). To our knowledge, no fungal records or herbal medicine like that described in Liu (1978) occur in Sir Lanka. Moreover, based on the examination of type material of *Hypocrea bambusae*, it has smaller (0.1 cm vs. 0.7–1.5 mm in diam.) and black stromata, unitunicate asci and ascospores are in a single fascicle but not significantly helically coiled (Fig. 7). Hence, we conclude that Liu (1978) made a wrong identification.

New collections of “Zhuhongjun” were collected and sequenced. The phylogenetic analyses showed it belongs to Shiraiaceae and is separate from *Shiraia* with high bootstrap support (100/1.00 MLBS/BSPP) (Fig. 2). *Grandigallia* has not been included in the phylogenetic tree as it is lacking gene sequences in the GenBank (retrieved date: 13 May 2019). However, *Grandigallia* can be morphologically distinguished from the new taxon in having black ascostromata and muriform ascospores (Barr 1987; Ariyawansa et al. 2013). Thus, this fungus is introduced as *R. bambusae* gen. et sp. nov in this study.

*Rubroshiraia bambusae* is often confused with *S. bambusicola* by Chinese traditional folk residents, probably because of the similarity of their ascostromata, parasitism on bamboo host and similar efficacy of medical treatment. However, it differs from *S. bambusicola* by its smaller sized ascostromata (0.7–1.2 cm long × 0.7–1 cm wide vs. 1–6 cm long × 1–4 cm wide) and distinct ascospores (filiform ascospores vs. fusiform and muriform ones). Both of the above species can produce the metabolites hypocrellin A and B, whereas *R. bambusae* contains almost double the content compared to *S. bambusicola* (Fig. 4).

### Rubroshiraia bambusae

Taxon classificationFungiPleosporalesShiraiaceae

D.Q. Dai & K.D. Hyde sp. nov.

B8CC7A3EDFC3517F8169748C3DCE32BA

Fig. 7

#### Etymology.

Refers the bamboo host.

#### Holotype.

HKAS102255.

#### Description.

**Parasitic** on living branches of bamboo. **Sexual morph**: ***Ascostromata*** 0.7–1.5 cm long × 0.7–1.3 cm wide, solitary, superficial, globose to subglobose, fleshy, reddish, with locules lining the periphery, with dark ostiolate points appearing on the surface. ***Ascostromatic tissue*** thick, pinkish, composed of wider woven hyphae of textura intricata. ***Locules*** in vertical section 800–1800 µm high × 1000–2000 µm diam. (*x̄* = 1289.4 ×1368.8 µm, n = 20), globose to subglobose, immersed in the periphery layer of ascostromata, with 250–500 µm wide × 450–550 µm high ostioles. ***Peridium*** 20–35 µm thick, composed of several layers of hyaline to dark brown, small cells of textura angularis to textura intricata. ***Hamathecium*** of interthecial, hyaline septate, branched pseudoparaphyses, 1–3 µm wide. ***Asci*** 660–800 × 45–55 µm (*x̄* = 751.6 × 49.5 µm, n = 20), 8-spored, thick-walled, bitunicate, fissitunicate, cylindrical, short-pedicellate, with an ocular chamber. ***Ascospores*** 600–750 × 5.5–11 µm (*x̄* = 728.8 × 9.1 µm, n = 20), spirally arranged in asci, filiform, hyaline, with 15–18 transverse septa, smooth-walled. **Asexual morph**: Undetermined.

#### Material examined.

CHINA, Yunnan, Dali, on living branches of *Fargesia spathacea* Franch, 13 May 2017, Dong-Qin Dai, DDQ00411 (HKAS102255, **holotype**), *Ibid*. (HMAS 290447, **isotype**), *Ibid*. DDQ00412 (HKAS102256), *Ibid*. DDQ00416 (HKAS102260), *Ibid*. 20 June 2017, Dong-Qin Dai, DDQ00425 (HKAS102268), *Ibid*. DDQ00426 (HKAS102269), *Ibid*. DDQ00427 (HKAS102270), *Ibid*. DDQ00428 (HKAS102271), *Ibid*. DDQ00429 (HKAS102272), *Ibid*. DDQ00430 (HKAS102273), *Ibid*. DDQ00431 (HKAS102274).

### Key for distinguishing genera in Shiraiaceae

**Table:** 

1	Parasitising bamboo branches, ascostromata are white to reddish	**2**
–	Parasitising Rosaceae branches, ascostromata are black	*** Grandigallia ***
2	Ascospores muriform	*** Shiraia ***
–	Ascospores filiform	*** Rubroshiraia ***

Since the familial placement of *H. bambusae* is controversial in different studies (Berkeley and Broome 1875, Saccardo 1878, Liu 1978), we re-studied the isotype. Based on morphology, we conclude that it has unitunicate asci thus related to Sordariomycetes.

### Hypocrella bambusae

Taxon classificationFungiPleosporalesShiraiaceae

(Berk. & Broome) Sacc. 1878

C63A90F9A247512CBE1F4AC911BD2CD0

Fig. 8

#### Basionym.

*Hypocrea bambusae* Berk. & Broome, 1873

#### Description.

**Parasitic** on living inflorescence of bamboo. **Sexual morph**: ***Stromata*** around 0.14 cm diam., 0.06 cm high, solitary, superficial, subglobose, fleshy to coriaceous, black, with around 20 perithecia lining the periphery, with ostioles slightly raised above stroma surface. ***Stromatic tissue*** thick, brown to dark brown. ***Perithecia*** in vertical section around 100 µm diam., 200 µm high, pyriform, immersed in the periphery layer of stromata. ***Asci*** more than 220 µm long, 5–6 µm diam., 8-spored, unitunicate, cylindrical, with a glassy refractive cap around 3 µm from apex to base. ***Ascospores*** around 180 µm long, 1–1.5 µm diam., in a single fascicle but not significantly helically coiled, filiform, hyaline, with 9–10 transverse septa, with rounded ends, smooth-walled. **Asexual morph**: Undetermined.

**Figure 8.**
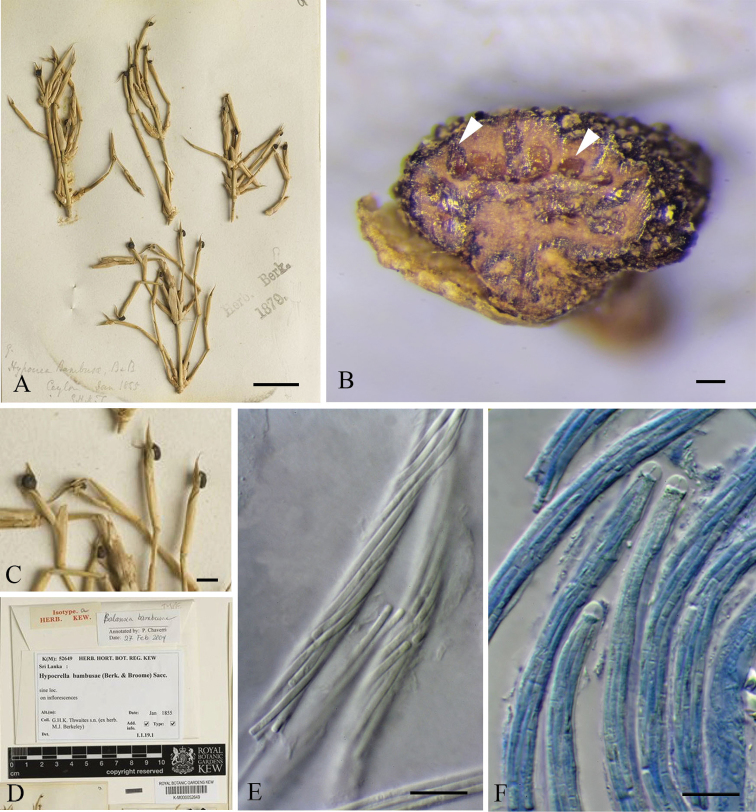
*Hypocrella bambusae* (K(M)52469, isotype, images are accredited to the Royal Botanic Gardens, Kew) **A, C** fruiting bodies on inflorescence of bamboo **B** vertical section of stromata showing the perithecia locating **D** herbarium envelope **E** filiform ascospores **F** asci with caps (Staining by cotton blue). Scale bars: 5 mm (**A**), 200 μm (**B**), 2 mm (**C**), 20 μm (**E, F**).

#### Material examined.

SRI LANKA, on inflorescence of bamboo, January 1855, G.H.K. Thwaites s.n. (ex herb. M.J. Berkeley), K(M)52469, **isotype**.

#### Notes.

This taxon has typical morphology of the *Clavicipitaceae*, which is pyriform perithecia with a gradually tapering upper part and cylindrical asci with a glassy refractive cap. New collections are required and need to be sequenced to clarify its placement.

## Discussion

Members of the family Shiraiaceae are distributed from Asia to South America but so far reported only from three countries, viz. China, Japan and Venezuela (Barr et al. 1987; Liu et al. 2013). The family comprises three genera, i.e. *Grandigallia*, *Rubroshiraia* and *Shiraia* wherein the former genus is lacking DNA sequences and, thus in here, we did not include it in the molecular analyses (Figs 2 and 3). These genera show the typical characters of Shiraiaceae, viz. conspicuous large, tuberculate, fleshy and multi-loculate ascostromata producing bitunicate asci. *Shiraia bambusicola* has various types of ascostromata, such as subglobose to tuberculate with white to pinkish colours (Fig. 6). However, the phylogenetic analysis shows these specimens with different types of ascostromata belong to same species (Figs 2 and 3). Thus, we assume that the different shapes of ascostromata are because of the host and different environment conditions.

Stromatal methanol extracts of *Rubroshiraia* and *Shiraia* contain Hypocrellins (Fig. 4). However, so far no extracts have been reported from *Grandigallia*. Fresh material of *Grandigallia* is essential to determine the metabolites. *Rubroshiraia* has darker reddish ascostromata compared with *Shiraia*, probably because its stromatal methanol extracts contain larger quantity of hypocrellins. Some endophytes, named as Shiraia-like fungi, are known to produce hypocrellins on media. They were isolated from different parts of bamboo, such as seeds, nods and internodes (Lu et al. 2004; Morakotkarn et al. 2008; Liang et al. 2009; Cai et al. 2011; Zhang et al. 2014). Other Shiraia-like endophytes, isolated from the rhizome of *Gastrodia*, leaves of *Huperzia serrata* and from *Triticum aestivum*, phylogenetically cluster within the former group (Fig. 3). However, no hypocrellins were produced from their mycelium (Zhu et al. 2010; Wang et al. 2011, 2016). The bamboo tissue may be providing the needful substances for fungi to produce hypocrellins. The endophytic Shiraia-like taxa (Fig. 3) appear as a distinct genus in Shiraiaceae. The nomination will be made once the type material is available.

*Shiraia bambusicola* has been used as a Chinese traditional folk-medicine, in curing rheumatoid arthritis, infantile convulsion and pertussis etc. for more than 400 years, because of its stromatal metabolites (Huang et al. 2001; Shen et al. 2002). Japanese scientists first obtained three perylenequinones from air-dried ascostromata of *S. bambusicola* and named them as hypocrellin A, B and C (Kishi et al. 1991). However, hypocrellin A was originally discovered by Wan and Chen (1981) from a different fungus on bamboo which was called as “Zhuhongjun” in Chinese and was erroneously identified as *H. bambusae* (Liu 1978). Later the fourth hypocrellin analogue (hypocrellin D) was named by Fang et al. (2006). Therefore, in total, four hypocrellins have so far been named. Hypocrellins are types of biologically active compounds and naturally occurring perylenequinones with photodynamic activity (Wan and Chen 1981; Kishi et al. 1991; Chowdhury et al. 2002; Liang et al. 2009; Liu et al. 2013). These secondary metabolites have gained much attention owing to their light-induced anti-tumour, anti-fungal and anti-viral activities (Wan and Chen 1981; Liang et al. 2009; Li et al. 2000a, b). In clinical trials, hypocrellin shows promising treatment for various skin diseases, such as skin cancer and white lesions of the vulva (Wan and Chen 1981; Li et al. 2000b). In China, a costly medicinal unguent named Bamboo Parasitic Fungus Ointment is made of hypocrellin B (Dai et al. 2018). Interestingly, it was proved that hypocrellin has bactericidal activities which inhibit various bacteria, such as *Bacillus subtilis* Ehrenberg and *Micrococcus luteus* Schroeter (Chen et al. 2010). In addition, hypocrellin A has an antiviral activity against human immunodeficiency virus (HIV-1) (Hudson et al. 1994) and is promising as a new-fashioned photoelectric conversion material (Li et al. 2000a).

Hypocrellin has wide application prospects, but it was earlier only found existing in ascostromata of *S. bambusicola* and “Zhuhongjun” (*R. bambusae* in this paper) (Wan and Chen 1981; Kishi et al. 1991). For gaining a high yield of Hypocrellin, scientists devoted themselves to looking for strains that can produce hypocrellin through fermentation production (Liang et al. 2009). Numerous endophytes, isolated from bamboo tissue such as culms, leaves, nodes and seeds, were published (Lu et al. 2004; Morakotkarn et al. 2007, 2008; Liang et al. 2009; Cai et al. 2011; Shen et al. 2012, 2014; Zhang et al. 2014), several of which had the potential for hypocrellin production (Lu et al. 2004; Morakotkarn et al. 2008; Liang et al. 2009; Zhang et al. 2014; Tong et al. 2017). However, the strains with promising industrial fermentation were identified as *Shiraia* sp. based on the blast search in GenBank by ITS sequences. More endophytes producing biologically active compounds, such as huperzine, isolated from the plant *Huperzia serrata* (Thunb. ex Murray) Trev., were also named as *Shiraia* sp. (Wang et al. 2011, 2016; Zhu et al. 2010). These strains usually have around 80%–90% ITS similarity with *S. bambusicola*, which also shows that they are phylogenetically close with members of Shiraiaceae. In this study, these endophytes are placed in Shiraiaceae, based on the phylogenetic analyses (Fig. 3).

According to Deng et al. (2017), polyketide synthase (*SbaPKS*) is involved in hypocrellin biosynthesis, based on the methods of CRISPR/Cas9 genome editing. It provides evidence for decoding the hypocrellin pathway (Deng et al. 2017). This pathway has the potential for producing high quality hypocrellins.

## Supplementary Material

XML Treatment for Shiraiaceae

XML Treatment for Shiraia bambusicola

XML Treatment for Grandigallia

XML Treatment for Rubroshiraia

XML Treatment for Rubroshiraia bambusae

XML Treatment for Hypocrella bambusae
